# Sequential Organ Failure Assessment (SOFA) scores differ between genders in a sepsis cohort: Cause or effect?

**DOI:** 10.3109/03009734.2012.703255

**Published:** 2012-10-30

**Authors:** Sofie Jacobson, Eva Liedgren, Göran Johansson, Martin Ferm, Ola Winsö

**Affiliations:** ^1^Department of Surgical and Perioperative Sciences, Anesthesiology and Intensive Care Umeå University, Umeå, Sweden; ^2^Research unit, Jämtland County, Östersund, Sweden

**Keywords:** APACHE II, gender, ICU length of stay, mortality, septic shock, severe sepsis, SOFA

## Abstract

**Background.:**

Controversy exists regarding the influence of gender on sepsis events and outcome. Epidemiological data from other countries may not always apply to local circumstances. The aim of this study was to identify gender differences in patient characteristics, treatment, and outcome related to the occurrence of sepsis at admission to the ICU.

**Methods.:**

A prospective observational cohort study on patients admitted to the ICU over a 3-year period fulfilling sepsis criteria during the first 24 hours. Demographic data, APACHE II score, SOFA score, TISS 76, aetiology, length of stay (LOS), mortality rate, and aspects of treatment were collected and then analysed with respect to gender differences.

**Results.:**

There were no gender-related differences in mortality or length of stay. Early organ dysfunction assessed as SOFA score at admission was a stronger risk factor for hospital mortality for women than for men. This discrepancy was mainly associated with the coagulation sub-score. CRP levels differed between genders in relation to hospital mortality. Infection from the abdominopelvic region was more common among women, whereas infection from skin or skin structures were more common in men.

**Conclusion.:**

In this cohort, gender was not associated with increased mortality during a 2-year follow-up period. SOFA score at ICU admission was a stronger risk factor for hospital mortality for women than for men. The discrepancy was mainly related to the coagulation SOFA sub-score. Together with differences in CRP levels this may suggest differences in inflammatory response patterns between genders.

## Introduction

A common observation in epidemiological studies of sepsis/severe sepsis is that men account for approximately 60% of patients, but the impact of gender on outcome is less clear (1–5). Although animal studies show an advantage in survival from a sepsis challenge for female mice (6,7), this observation is not consistently supported by human studies. Gender-related differences in immune response have been ascribed to the influence of sex hormones as well as genetic polymorphism (8,9). A French study showed that the overall hospital mortality from severe sepsis was lower in women and that this discrepancy was due to lower mortality in post-menopausal women (10). Other studies have reported a higher incidence of sepsis in men but no gender-related differences in mortality (11–13). However, there are reports of higher case fatality rates among women suffering from sepsis (14), of female gender being an independent predictor of increased mortality in patients with documented infection (4), and studies of mainly surgical patients report a poorer outcome from severe sepsis for women (15,16).

A German study found no difference in intensive care unit (ICU) mortality between genders in a large cohort of ICU patients, but in the subgroup of patients with sepsis the probability for ICU mortality was nearly twice as high for women (16). In a recent study on patients with severe sepsis or septic shock, Pietropaoli et al. found that women had a higher risk of dying in hospital. They found differences in delivery of care between genders, but these disparities did not explain the higher mortality in women. After multivariable adjustment the likelihood for hospital mortality was approximately 10% higher for women (17). Thus, there are discrepancies in study results regarding gender-related differences in the occurrence and outcome of sepsis. The impact of case mix, ethnicity, socio-economic factors, and local therapeutic traditions on the results from different studies is not easy to assess. We therefore conducted this study with the aim to investigate the occurrence of sepsis within 24 hours after admission to the ICU, with special attention to gender-related differences in patient characteristics, treatment, and outcome.

## Material and methods

This is a prospective observational cohort study, regarding analysis of data from septic patients admitted to the ICU of Umeå University Hospital, from 1 January 2003 to 31 of December 2005. The study was approved by the ethical committee at Umeå University. Umeå University Hospital is an 800-bed hospital in Northern Sweden, with a tertiary referral population of approximately 900,000. The ICU is a multidisciplinary, 14-bed unit.

Patients eligible for the study were identified by daily reviews of patient charts during weekdays. Data were retrieved from medical records, hospital mainframe computer, and patient data management system (Picis, Dräger Medical, Sweden AB). Inclusion criteria were age ≥18 years and an admission diagnosis of sepsis or development of severe sepsis or septic shock within 24 hours after ICU admission, according to standard definitions (18). Sequential Organ Failure Assessment (SOFA) score as a marker for organ dysfunction and disease severity was calculated at admission (SOFA_0) and then daily for the first 14 days and on the day of discharge, if after the initial 14 days (19). The daily SOFA score was based on the worst value during each 24-hour period from 06.00 to 06.00. SOFA_max was defined as the maximum value of the SOFA score during the ICU stay. A SOFA organ sub-score of 2 was considered a sign of organ dysfunction and a SOFA organ sub-score of 3 or more was considered a sign of organ failure.

APACHE II was used for assessment of severity of illness (20).

Therapeutic Intervention Scoring System (TISS) 76 was registered once daily and used as an estimate of personnel work-load (21).

Patients were characterized according to referral pattern, reason for admission, co-morbidities, source of infection, primary infection site, and infection-causing micro-organism. Microbiological cultures were considered relevant only if acquired within 48 hours before or after admission to the ICU. Aspects of treatment included cardiovascular, respiratory, and renal support, transfusions, administered nutritional solutions and other fluids, sedation, antibiotics and medication relevant for the treatment of sepsis, as well as surgical interventions. Mechanical ventilation was defined as ventilation of patients who were endotracheally intubated or tracheostomized. Antibiotics, antifungal, and antiviral drugs were registered and grouped in accordance with the anatomic therapeutic chemical classification system (ATC). Pre-existing diseases were defined as described by Knaus et al. (20). Data on hospital length of stay and hospital mortality were obtained from the hospital record system. Two-year follow up mortality was obtained from a national database.

To assure data quality a second evaluation of each patient's data was conducted by one of the authors not responsible for the primary data collection. For patients admitted more than once to the ICU, only the first sepsis-related admission was included.

### Statistics

Data were collected on a spreadsheet (Microsoft Excel). For statistical analysis, SPSS v. 19 (SPSS, Inc., Chicago IL, USA) was used. Data are presented as numerical values or percentages for categorical variables, and mortality rates are presented as proportions with 95% confidence intervals (CI). Continuous data are presented as mean with standard deviation, or median and first and third quartiles, according to distribution. For statistical comparisons between gender, depending on sample size, Pearson chi-square tests or Fisher's exact test were used for categorical values and for continuous variables two-tailed *t* test or Mann–Whitney *U* test according to proof of normality. Odds ratios (OR) are reported with 95% CI. A *P* value < 0.05 was considered significant, and all *P* values reported are two-sided. No adjustment was made for multiple testing.

Univariate logistic regression was performed to evaluate independent risk factors for hospital mortality. Covariates were all variables in Table I: scorings (APACHE II, TISS 76, SOFA scores), septic shock, maximum lactate level during first 24 hours, and maximum creatinine and C-reactive protein (CRP) level during ICU stay.

**Table I.** Patient characteristics.

		Women	Men	
		*n* = 50	*n* = 77	
		No (%)	No (%)	*P*
Referral pattern	Admission from the community	6 (12)	10 (13)	ns
	ICU transfer from within hospital	35 (70)	54 (70)	ns
	Transfer from other institution	9 (18)	13 (17)	ns
Patient category	Medical^a^	28 (56)	59 (76)	0.019
	Surgical elective	6 (12)	9 (12)	ns
	Surgical emergency^a^	16 (32)	9 (12)	0.006
Co-morbidities	Congestive heart failure	3 (6)	3 (4)	ns
	Chronic lung disease	4 (8)	4 (5)	ns
	Chronic liver disease	0 (0)	3 (4)	ns
	Chronic renal insufficiency	1 (2)	5 (6)	ns
	Diabetes	11 (22)	16 (21)	ns
	Cancer			
	Haematological	5 (10)	8 (10)	ns
	Localized	9 (18)	9 (12)	ns
	Metastatic	3 (6)	3 (4)	ns
	Immunosuppressants			
	Chronic steroids	3 (6)	6 (8)	ns
	Chemotherapy	7 (14)	8 (10)	ns
	Radiotherapy	4 (8)	2 (3)	ns
	Other immunosuppression	2 (4)	5 (6)	ns
	Other chronic disabling conditions	7 (14)	19 (25)	ns
Number of co-morbidities	0	19 (38)	22 (28)	ns
	1	11 (22)	29 (38)	ns
	2	9 (18)	18 (23)	ns
	>3	11 (22)	8 (10)	ns
Infection characteristics	Community-acquired	35 (70)	58 (75)	ns
	Nosocomial	15 (30)	19 (24)	ns
Primary infection site	Pneumonia	10 (20)	17 (22)	ns
	Abdominopelvic^a^	17 (34)	8 (10)	0.002
	Urinary tract	3 (6)	14 (18)	ns
	Other	14 (28)	23 (30)	ns
	Skin or skin structures^a^	0 (0)	9 (12)	0.012
	Indwelling catheter	4 (8)	3 (4)	ns
	Unknown	2 (4)	3 (4)	ns
Micro-organism	Gram-positive cocci	18 (36)	27 (35)	ns
	Gram-positive rods	0 (0)	2 (3)	ns
	Gram-negative rods	11 (22)	19 (24)	ns
	Fungi	6 (12)	8 (10)	ns
	Other	3 (6)	7 (9)	ns
	Mixture	5 (10)	5 (6)	ns

Other chronic disabling conditions include patients with Myelomeningocele and urinary bladder dysfunction, patients with tetraplegia of various underlying causes, patients with inflammatory bowel disease, and patients with multiple diseases other than those defined above. Other immunosuppressant includes azathioprine, ciclosporin, and TNF-α inhibitors. Community-acquired defined as infection developed within 48 hours after hospital admittance. Type of micro-organism retrieved from cultures from blood, urine, cerebrospinal fluid, synovial fluid, pleural fluid, and tissues.

^a^Statistically significant difference between genders.

An interaction analysis was performed between the independent variables and gender, with hospital death as outcome variable. Organ sub-scores for SOFA_0 and SOFA_1 were included in this analysis.

A multivariate backward stepwise logistic regression adjusted for age and gender was performed with hospital mortality as dependent variable. Independent variables were variables from the univariate logistic regression analysis with a *P* value of < 0.05. APACHE II, SOFA_0, and SOFA_max were entered separately in the regression model, and all were statistically significant. Colinearity existed between the APACHE II score and SOFA scores. Only SOFA_0 was used in the final model.

## Results

### Demographic data

During this 3-year period, 2271 patients (1388 men and 883 women) were admitted to the ICU, with a mean ICU length of stay of 4.1 days and an overall ICU mortality of 8.5%. Of the 2271 patients, 127 patients fulfilled the inclusion criteria for severe sepsis or septic shock during the first 24 hours after ICU admission. Of the 127 patients, 60% were men. Patient characteristics, referral patterns, and admission categories are presented in Table I. Women were significantly more often admitted after emergency surgery (*P* = 0.006) and men significantly more often due to medical reasons (*P* = 0.019).

### Infection characteristics

The majority of patients had a community-acquired infection (73%). There was a significant gender-related difference in source of infection. Infection from the abdominopelvic region was significantly more common in women (*P* = 0.002), while sepsis originating from skin or skin structures was significantly more common in men (*P* = 0.012). Positive blood cultures were obtained from 52 patients (41%). In 37 patients (29%) cultures other than from blood were positive, while in 38 patients (30%) microbiological cultures, including blood, urine, liquor, wound, airway, and others, were negative. Ten patients, 5 men and 5 women, had fungi as single infecting micro-organism. No patient had multi-resistant bacteria as primary infecting agent. There were no differences between men and women regarding proportion of positive microbiological cultures, or infection with Gram-positive, Gram-negative bacteria, or fungi (Table I).

### Treatment

Of the treatment modalities presented in Table II, there were no differences between genders regarding frequency or duration of treatment, except that women received surgical drainage more often. Of the patients not endotracheally intubated, all had intermittent respiratory support via face mask, CPAP or Bi-level support. Renal replacement therapy was needed in 27 (21%) patients, of whom 5 patients had only intermittent haemodialysis (HD) and 22 patients had HD and/or continuous renal replacement therapy. Cardiovascular monitoring with echocardiography performed by specially trained echo-cardiographers or pulse contour intermittent thermodilution cardiac output monitoring technique (PiCCO, Pulsion Medical Systems AG, Munich, Germany) was used to a similar extent in men and women.

**Table II.** Treatment, fluids, and antibiotics.

A. Treatment			Women *n* = 50	Duration (days)	Men *n* = 77	Duration (days)	
Modality			No (%)	Median (25/75 percentile)	No (%)	Median (25/75 percentile)	*P*
Vasopressor support			37 (74)	5.0 (2.0/6.5)	55 (71)	3 (2.0/7.0)	ns
Endotracheally intubated			34 (68)	7.5 (4.0/8.0)	48 (62)	8 (4.0/8.0)	ns
CRRT/HD			12 (24)	7.0 (5.0/8.0)	15 (19)	8 (3.0/8.0)	ns
Low-dose steroids			28 (56)	6.5 (4.0/8.0)	41 (53)	7 (2.0/8.0)	ns
Sedation			36 (72)	8.0 (4.0/8.0)	51 (66)	7 (3.0/8.0)	ns
Parenteral nutrition			37 (74)	5.0 (2.0/8.0)	52 (68)	5 (2.0/8.0)	ns
Enteral nutrition			37 (74)	5.0 (2.5/8.0)	56 (73)	6 (2.0/8.0)	ns
Platelet transfusion			12 (24)	2.5 (1.0/4.0)	19 (25)	1 (1.0/3.0)	ns
Low-molecular-weight heparin			39 (78)	7.0 (3.0/8.0)	56 (73)	7 (3.0/8.0)	ns
Surgical procedures^a^			25 (50)		24 (31)		0.041
Monitoring							
Echocardiography			27 (54)		43 (56)		ns
PiCCO			14 (28)		19 (25)		ns
**B. Fluids**			**Women *n* = 50**	**Volume (L)**	**Men *n* = 77**	**Volume (L)**	
**Type of fluid**			**No (%)**	**Median (25/75 percentile)**	**No (%)**	**Median (25/75 percentile)**	***P***
Total volume at 2 hours			43 (86)	2.0 (1.0/2.5)	63 (82)	2.0 (1.0/3.5)	ns
24 hours							
Crystalloids			44 (88)	2.6 (1.0/4.0)	71 (92)	3.0 (2.0/5.0)	ns
Human albumin 5%			38 (76)	1.0 (0.5/1.6)	45 (68)	1.0 (0.8/1.5)	ns
Human albumin 20%			27 (64)	0.2 (0.1/0.4)	46 (60)	0.2 (0.1/0.4)	ns
Synthetic colloids			21 (42)	1.0 (0.5/1.0)	30 (39)	1.0 (0.5/1.5)	ns
Total volume at 24 hours			48 (96)	4.0 (2.0/5.7)	77 (100)	3.8 (2.5/6.2)	ns
Fresh frozen plasma			24 (48)	1.0 (0.5/1.7)	35 (44)	1.0 (0.5/1.5)	ns
Red blood cells^a^			36 (72)	0.8 (0.6/1.2)	41 (53)	0.6 (0.6/1.2)	0.042
ICU-LOS							
Human albumin 5%			46 (92)	2.2 (1.0/3.7)	64 (83)	2.2 (1.0/3.8)	ns
Human albumin 20%			39 (78)	0.5 (0.3/1.2)	55 (71)	0.5 (0.2/1.2)	ns
Synthetic colloids			24 (48)	0.9 (0.5/1.4)	42 (54)	1.0 (0.5/2.0	ns
Fresh frozen plasma			29 (58)	1.5 (1.0/3.8)	42 (54)	1.8 (0.5/3.3)	ns
Red blood cells			40 (80)	1.4 (0.8/2.3)	52 (67)	1.2 (0.6/2.6)	ns
Platelets			12 (24)	0.8 (0.3/1.4)	19 (25)	0.6 (0.3/1.2)	ns
**C. Antibiotics**			**Women *n* = 50**	**Administered doses (*n*)**	**Men *n* = 77**	**Administered doses (*n*)**	
**Type of antibiotic**			**No (%)**	**Median (25/75 percentile)**	**No (%)**	**Median (25/75 percentile)**	***P***
Meropenem			23 (46)	11 (6/28)	42 (54)	17 (9/24)	ns
Ciprofloxacin			21 (42)	9 (2/15)	29 (38)	10 (4/14)	ns
Piperacillin/tazobactam			20 (40)	16 (6/33)	28 (36)	10 (5/24)	ns
Cefuroxime^a^			11 (22)	9 (4/23)	16 (21)	1 (1/4)	0.025
Clindamycin			8 (16)	11 (3/41)	19 (25)	18 (8/33)	ns
Vancomycin/teicoplanin			9 (18)	6 (3/15)	18 (23)	3,5 (1/13)	ns
Aminoglycosides			11 (22)	8 (1/14)	15 (19)	3 (2/4)	ns
Cefotaxim/ceftazidim			6 (12)	18 (9/25)	13 (17)	12 (3/23)	ns
Ampicillin^a^			5 (10)	9 (9/18)	10 (13)	6 (3/9)	0.036
Antimycotics (J02A)			20 (40)		29 (38)		ns

Surgical procedures include removal of gastrointestinal, biliary, and urinary obstructions; debridement; drainages of abscesses, pleural space, joints, and surgical drainages^a^.

Crystalloids defined as Ringer's acetate and isotonic NaCl; synthetic colloid solutions include hydroxyethyl starch 130/0.4 6%, hydroxyethyl starch 200/0.5 6%, and dextran 70, 6%; blood products include packed red blood cells, fresh frozen plasma, and platelets. Total volumes at 2 and 24 hours defined as fluid administered for purpose of volume substitution (maintenance drip, nutritional solutions, infusions, and blood products excluded). Aminoglycosides include netilmicin, amikacin, gentamicin. Benzyl penicillin and tetracycline are omitted from the table; for information see text.

^a^Statistically significant difference between genders.

CRRT = continuous renal replacement therapy; HD = haemodialysis; LOS = length of stay; PiCCO = pulse contour intermittent thermodilution continuous cardiac output monitoring.

No patient was treated with Rh-APC. All patients were treated with parenteral antibiotics during the whole ICU length of stay. Regarding the choice of antibiotics there was no difference in proportion of administered treatment other than for tetracycline, which was administered to 5 men only, and benzyl penicillin, administered to 1 woman and 12 men. Women received significantly more cefuroxime (*P* = 0.025) and ampicillin (*P* = 0.036), compared to men (Table II).

Volumes of resuscitation fluids administered within 24 hours after ICU admission and during the whole ICU length of stay are summarized in Table II. Administration of fluids within 2 hours from admission to the ICU did not differ between genders. At 24 hours after ICU admission significantly more women than men had received transfusion with packed red blood cells (*P* = 0.042). There was no significant difference between men and women in total volumes of resuscitation fluids or blood products administered during the ICU stay (Table II).

### Outcome data

**Figure 1.**
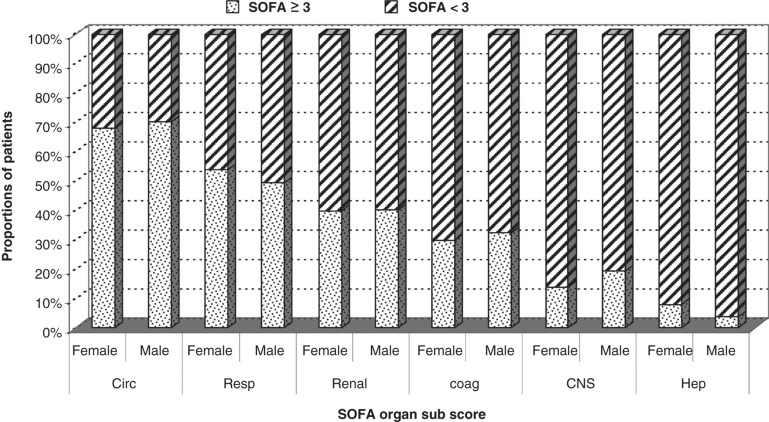
Proportion of patients with SOFA organ sub-score ≥3 as a sign of organ failure (females and males) and proportion of patients with SOFA organ sub-score <3 (females and males). SOFA sub-scores: circ = circulatory; resp = respiratory; renal = renal; coag = coagulation; CNS = central nervous system; hep = liver function.

There were no gender-related differences in mortality rates or length of stay (Table III). Concerning scoring, there were no significant differences between genders in total SOFA scores at admission, day 1, or maximum score (Table III). The proportions of men and women with SOFA organ sub-scores of 2, indicating organ dysfunction (data not shown), or sub-scores of 3 or more, indicating organ failure, were similar (Figure 1).

**Table III.** Scoring and outcome.

	Women *n* = 50		Men *n* = 77		
	Mean	SD	Mean	SD	*P*
Age (years)	61.3	15.66	63.3	13.63	ns
Scoring (point)					
APACHE II score	19.6	6.01	20.0	6.88	ns
SOFA_0	7.5	3.88	8.1	3.87	ns
SOFA_1	8.5	4.54	7.4	4.17	ns
SOFA_max	9.4	4.45	10.2	4.27	ns
TISS 76/ICU day	26.1	8.13	26.9	9.50	ns
LOS (days)	Median	25/75 percentile	Median	25/75 percentile	
ICU	8	(3/13.2)	6	(3/13)	ns
Survivors	7	(3.2/13.8)	8	(3.2/14.8)	ns
Non-survivors	8.5	(2/14.2)	3	(2/8.5)	ns
Hospital	24.5	(12/37)	17	(9/35)	0.055
Survivors	31	(15/64)	18	(12/35.5)	0.082
Non-survivors	18	(9/22)	8	(3/34)	ns
Mortality (%)	No (%)	95% CI	No (%)	95% CI	
ICU	10 (20)	0.11–0.33	17 (22)	0.14–0.33	ns
28 days	12 (24)	0.14–0.38	22 (29)	0.20–0.40	ns
Hospital	11 (22)	0.13–0.35	25 (32)	0.23–0.44	ns
3 months	12 (24)	0.14–0.38	30 (39)	0.29–0.50	ns
6 months	15 (30)	0.19–0.44	32 (42)	0.31–0.53	ns
1 year	21 (42)	0.29–0.56	35 (46)	0.35–0.57	ns
2 years	21 (42)	0.29–0.56	35 (46)	0.35–0.57	ns

SOFA_0 defined as SOFA at admission; SOFA_1 based on the highest values during the first whole 24-hour period from 06.00 to 06.00; and SOFA_max defined as the highest score during the ICU-LOS. APACHE II = Acute Physiology and Chronic Health Evaluation score; SOFA = Sequential Organ Failure score; TISS 76 = Therapeutic Intervention Scoring System (76 items); ICU = intensive care unit; LOS = length of stay.

**Figure 2.**
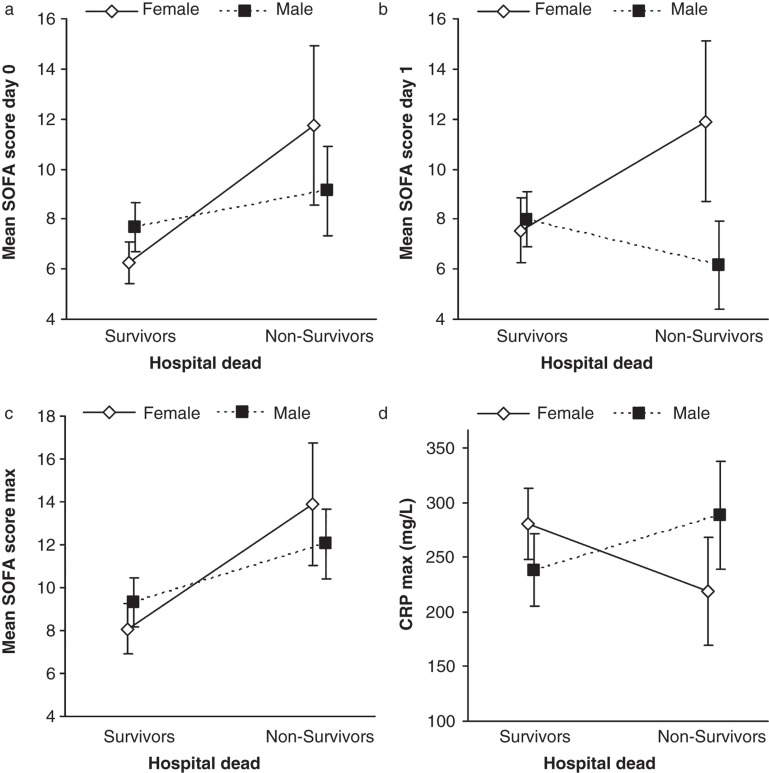
Differences between genders in SOFA scores and CRP max in relation to hospital outcome. Panel A: SOFA score at admission (SOFA_0) was significantly higher in non-surviving than surviving women (*P* = 0.001), but not among surviving compared to non-surviving men. Panel B: SOFA score day 1 (SOFA_1) was significantly higher among hospital non-surviving compared to surviving women (*P* = 0.008), but in men SOFA_1 was significantly lower in non-surviving men compared to surviving men (*P* = 0.035). Panel C: SOFA_max was significantly higher among hospital non-survivors compared to survivors in both women (*P* = 0.001) and men (*P* = 0.017). Panel D: The interaction between gender and CRP as a risk factor for hospital mortality. CRP_max was significantly lower in surviving women than in non-surviving women (*P* = 0.035). Men displayed a different pattern with higher CRP in surviving men than non-surviving men, although the difference was not statistically significant (*P* = 0.081). (CRP = C-reactive protein. Data are presented as mean ± 95% confidence intervals).

SOFA_0 and SOFA_1 were significantly higher in non-surviving compared to surviving women. Men differed in that respect: SOFA_0 was not significantly higher in non-surviving than in surviving men (Figure 2A), and SOFA_1 was significantly lower among non-surviving compared to surviving men (Figure 2B). SOFA_max was significantly higher among hospital non-survivors compared to survivors in women and men (Figure 2C).

APACHE II score was significantly higher among both non-surviving men and women compared to hospital survivors (women *P* = 0.029; men *P* = 0.010), but no difference was found between genders (data not shown).

TISS 76 scores differed neither totally nor per day between genders, regardless of outcome (data not shown).

### Predictors of mortality

Results from the univariate logistic regression analysis with hospital mortality as dependent variable are displayed in Table IV. Neither gender nor sources of infection or infective microbiological agents were identified as risk factors for hospital mortality. As opposed to other scores, total TISS scores or TISS scores/day were not associated with mortality.

**Table IV.** Analysis of risk factors for hospital death.

Univariate logistic regression analysis	Unadjusted odds ratio (95% CI)		
Background variables	OR	95% CI	*P*
Gender	1.70	(0.75–3.88)	0.203
Age (years)	1.05	(1.01–1.08)	0.007
Admission type medical	2.98	(1.13–7.90)	0.028
APACHE II	1.12	(1.05–1.20)	0.001
SOFA_0	1.22	(1.09–1.36)	< 0.001
SOFA_1^a^	1.01	(0.92–1.10)	0.883
SOFA_max	1.26	(1.13–1.41)	< 0.001
Haematological disease	7.25	(2.07–25.42)	0.002
Chronic corticosteroid medication	10.74	(2.11–54.62)	0.004
Septic shock	2.65	(1.11–6.31)	0.028
C-reactive protein^a^	1.00	(0.997–1.004)	0.621
	Unadjusted odds ratio (95% CI)		
Treatment modalities	OR	95% CI	*P*
Vasopressor support	1.13	(0.99–1.29)	0.076
Endotracheally intubated	0.98	(0.89–1.10)	0.781
CRRT/HD	2.86	(1.19–6.88)	0.019
Low-dose steroids	3.35	(1.42–7.91)	0.006
Sedation	0.99	(0.88–1.10)	0.825
Parenteral nutrition	0.93	(0.82–1.05)	0.241
Enteral nutrition	0.37	(0.16–0.85)	0.019
Platelet transfusion	1.46	(1.10–1.92)	0.008
Low-molecular-weight heparin	0.90	(0.80–1.01)	0.067
Surgical procedures	0.51	(0.22–1.19)	0.119
	Adjusted odds ratio (95% CI)		
Multivariate logistic regression analysis	OR	95% CI	*P*
Medical admission type	3.92	(1.18–12.99)	0.025
Chronic corticosteroid treatment	14.21	(1.90–106.44)	0.010
SOFA_0	1.25	(1.10–1.43)	0.001

^a^Factors with significant interaction with gender; see under Results and Figure 2 for further information. Unadjusted OR = univariate analysis with hospital death as dependent variable; adjusted OR = multivariate stepwise backward logistic regression analysis, adjusted for age and gender, hospital death as dependent variable.

A multivariate logistic regression analysis, adjusted for age and gender, showed admission type medical, chronic corticosteroid medication and SOFA_0 as significant risk factors for hospital death (Table IV).

Interaction analysis between gender and independent variables revealed significant interaction with SOFA_0 (*P* = 0.026) and SOFA_1 (*P* = 0.001) as implied in Figure 2A and B. Further analysis of SOFA organ sub-scores showed significant interaction between gender and SOFA_0 coagulation sub-score (*P* = 0.024), SOFA_1 coagulation sub-score (*P* = 0.002), and SOFA_1 renal sub-score (*P* = 0.003). Also, CRP at admission (*P* = 0.028) and maximal CRP (*P* = 0.016) during ICU length of stay showed a significant interaction with gender as a risk factor for hospital death. The effect of this interaction between gender and CRP on hospital mortality is illustrated in Figure 2D.

A univariate logistic regression was performed with each aspect of treatment as covariate in order to assess their association with hospital mortality (Table IV). Of the treatment modalities associated with hospital mortality, only number of days of platelet transfusion was significantly associated with hospital mortality when introduced in the multivariate analysis (OR 1.66, 95% CI 1.34–2.43, *P* = 0.009, adjusted for gender, age, SOFA_0, medical status at admission, and chronic corticosteroid treatment).

## Discussion

In this cohort of patients, we found disparities between men and women in the significance of early SOFA scores, as a risk factor for hospital mortality. SOFA score at admission or day 1 was a stronger risk factor for hospital mortality for women than for men. This discrepancy was significant despite no discernible differences in total SOFA scores or SOFA organ sub-scores between genders. The difference between genders in SOFA scores as a risk factor for mortality was mainly related to the coagulation sub-score, i.e. the platelet count. There were also discrepancies between genders in the pattern of CRP in relation to hospital mortality.

In the present study SOFA score was considered a measure of organ dysfunction as originally intended (15). SOFA score was developed as a mean to evaluate morbidity in septic patients over time, but not to predict mortality. Even so, many studies have reported a good to excellent ability of the SOFA score to discriminate between survivors and non-survivors in intensive care patients in general, and some studies have investigated the discriminative power of individual organ scores (22). A majority of studies evaluating differences between scoring systems, different derivatives of the SOFA score, and the significance of temporal development of organ dysfunctions have included mixed intensive care patients and not specifically patients with sepsis. Above all, the gender aspects have not been addressed in these studies, and the score itself does not take gender into account.

There is a growing body of evidence that thrombocytopenia is related to an adverse outcome in critically ill patients (23). Several studies have reported low platelet count as independently related to ICU mortality, both in patients with bloodstream infection (24) and in general ICU populations (25,26). Low initial platelet counts as well as a reduction during the ICU stay seem to increase the risk of death, but the aspect of gender is not explicitly evaluated.

In studies from the intensive care community, when reported, the proportion of men is often 60% or more (10,12,27,28). In terms of evaluation of risk factors or effects of treatments based on study cohorts, whether from a general population or from sepsis subgroups, the fact that a majority of intensive care patients are men is a source of concern. The predominance of male gender in study cohorts may abolish the effect of treatment or the effect of risk factors that actually exist in the female gender.

Site of infection may constitute a confounder regarding outcome of sepsis, and there are inconclusive data concerning the influence of site of infection on both mortality and length of stay (12,29). It has been stated that infections originating from the urinary tract are associated with a favourable outcome and that abdominal infections are associated with increased risk of ICU death (29). Whether this holds true between genders is not clear, but the urinary tract is more frequently reported as a source of sepsis in women (10,12,17). Crabtree and co-workers showed that women with infection from skin and skin structures had a higher hospital mortality rate than men (30). A common finding is that the lungs and abdomen are the most frequent sites of infection in ICU patients (1,5,31,32). A sub-analysis of the SOAP study on the impact of infection originating from the lungs or the abdomen found differences in patient profiles and hospital length of stay (LOS), but the mortality rate was identical (32). In that study, septic shock was more common at admission in patients with abdominal infection, and they were more likely to have early coagulation failure and acute renal failure. However, there was no analysis regarding differences related to gender.

Whether the difference between genders in early SOFA score, and especially coagulation sub-score, as a risk factor for mortality in the present cohort is related to differences in source of infection (with abdomen as the predominant focus for women and a pre-ICU admission insult in terms of emergency surgery) or differences in the primary inflammatory response (where platelets play different roles in men and women) remains to be elucidated. Differences between genders in CRP levels in relation to outcome may also represent a part of a gender-related inflammatory response pattern or be related to differences in source of infection.

There were minor discrepancies in the treatment of men and women, but none of these treatment modalities were associated with mortality. The only treatment modality associated with mortality in the multivariate analysis was transfusion of platelets which was evenly distributed between genders. Specifically, mortality was not related to the total amount administered but the number of days that platelet transfusion was required. However, since a treatment is instituted because of a condition or an underlying disease it can be disputable to consider treatment per se as a risk factor. In line with a recent study, volume of resuscitation fluids and treatment with vasopressors were not significantly associated with hospital mortality (33).

Differences in antibiotic treatment were attributed to differences in sources of infection. All patients were considered to have adequate antibiotic treatment, in accordance with national guidelines, within 24 hours from ICU admittance. Time to first dose of antibiotics was not considered in the prospective data collection, and it proved to be difficult to obtain robust data retrospectively, since in a majority of the patients antibiotic treatment was already instituted before ICU admission.

We did not detect any statistically significant difference in mortality during a 2-year follow-up period between genders. Men had higher mortality rates in hospital, at 3 and 6 months, but, as stated above, these discrepancies were not statistically significant. The mortality rates are in line with recent Scandinavian studies (34,35). There was no statistically significant difference in ICU or hospital LOS between genders, but a tendency for longer hospital LOS among women, even when comparing survivors only.

This study represents the standard of care of an unselected patient population at a university hospital. From a socio-economic and ethnical perspective this cohort represents a homogeneous group of patients which reduces the influence of these factors on outcome. No specific intervention was made before the start of this observational study. The study was planned and data collection started before the guidelines from the Surviving Sepsis Campaign were published, thus the aim was not to study adherence to specific bundles or protocols.

To conclude, gender was not associated with increased hospital mortality. SOFA score at ICU admission and day 1 was a stronger risk factor for mortality for women. The discrepancy was mainly related to the coagulation SOFA sub-score. There were also differences between genders in CRP levels in relation to hospital mortality. Whether this discrepancy represents a gender-related difference in inflammatory response or is a consequence of differences in source of infection, or differences in time to institution of care, remains to be further elucidated.
